# Fibronectin inhibitor pUR4 attenuates tumor necrosis factor α–induced endothelial hyperpermeability by modulating β1 integrin activation

**DOI:** 10.1186/s12929-019-0529-6

**Published:** 2019-05-16

**Authors:** Ting-Hein Lee, Sung-Tsang Hsieh, Hou-Yu Chiang

**Affiliations:** 1 Department of Anatomy, College of Medicine, Chang Gung University, 259 Wenhua 1st Rd., Guishan Dist, Taoyuan City, 33302 Taiwan; 2 Graduate Institute of Biomedical Sciences, College of Medicine, Chang Gung University, Taoyuan, Taiwan; 3 Division of Cardiology, Department of Internal Medicine, Chang Gung Memorial Hospital, Linkou, Taiwan; 4 Department of Anatomy and Cell Biology, College of Medicine, National Taiwan University, Taipei, Taiwan; 5 Graduate Institute of Brain and Mind Sciences, College of Medicine, National Taiwan University, Taipei, Taiwan; 6 Department of Neurology, National Taiwan University Hospital, Taipei, Taiwan

**Keywords:** Endothelial permeability, Fibronectin, β1 integrin, Stress fiber

## Abstract

**Background:**

The blood–spinal cord barrier (BSCB) is composed of a monolayer of endothelium linked with tight junctions and extracellular matrix (ECM)-rich basement membranes and is surrounded by astrocyte foot processes. Endothelial permeability is regulated by interaction between endothelial cells and ECM proteins. Fibronectin (FN) is a principal ECM component of microvessels. Excessive FN deposition disrupts cell–cell adhesion in fibroblasts through β1 integrin ligation. To determine whether excessive FN deposition contributes to the disruption of endothelial integrity, we used an in vitro model of the endothelial monolayer to investigate whether the FN inhibitor pUR4 prevents FN deposition into the subendothelial matrix and attenuates endothelial leakage.

**Methods:**

To correlate the effects of excessive FN accumulation in microvessels on BSCB disruption, spinal nerve ligation—which induces BSCB leakage—was applied, and FN expression in the spinal cord was evaluated through immunohistochemistry and immunoblotting. To elucidate the effects by which pUR4 modulates endothelial permeability, brain-derived endothelial (bEND.3) cells treated with tumor necrosis factor (TNF)-α were used to mimic a leaky BSCB. A bEND.3 monolayer was preincubated with pUR4 before TNF-α treatment. The transendothelial electrical resistance (TEER) measurement and transendothelial permeability assay were applied to assess the endothelial integrity of the bEND.3 monolayer. Immunofluorescence analysis and immunoblotting were performed to evaluate the inhibitory effects of pUR4 on TNF-α-induced FN deposition. To determine the mechanisms underlying pUR4-mediated endothelial permeability, cell morphology, stress fiber formation, myosin light chain (MLC) phosphorylation, and β1 integrin–mediated signaling were evaluated through immunofluorescence analysis and immunoblotting.

**Results:**

Excessive FN was accumulated in the microvessels of the spinal cord after spinal nerve ligation; moreover, pUR4 inhibited TNF-α-induced FN deposition in the bEND.3 monolayer and maintained intact TEER and endothelial permeability. Furthermore, pUR4 reduced cell morphology alteration, actin stress fiber formation, and MLC phosphorylation, thereby attenuating paracellular gap formation. Moreover, pUR4 reduced β1 integrin activation and downstream signaling.

**Conclusions:**

pUR4 reduces TNF-α-induced β1 integrin activation by depleting ECM FN, leading to a decrease in endothelial hyperpermeability and maintenance of monolayer integrity. These findings suggest therapeutic benefits of pUR4 in pathological vascular leakage treatment.

**Electronic supplementary material:**

The online version of this article (10.1186/s12929-019-0529-6) contains supplementary material, which is available to authorized users.

## Background

The blood–spinal cord barrier (BSCB) of the microvessels in the spinal cord, which is similar to the blood–brain barrier (BBB) of the microvessels in the brain, constitutes a physical and biochemical barrier between the central nervous system (CNS) and systemic circulation [1]. The BSCB is composed of a monolayer of nonfenestrated capillary endothelium sealed with tight junctions, extracellular matrix (ECM)-rich basal lamina, and astrocyte foot processes [1]. The barrier function of the BSCB is strictly controlled by endothelial cell–cell tight junctions [2, 3]. Furthermore, cell–ECM adhesion via integrin ligation contributes to BSCB function [4, 5].

Integrins, a large family of transmembrane receptors for ECM proteins and cell-surface proteins [6–8], are heterodimers composed of two distinct subunits, α and β, both of which have a single-pass transmembrane domain [6–8]. In total, 18 α and 8 β chains associate through noncovalent bonding to form 24 distinct heterodimers with different ligand binding properties and distributions [7]. Ligation of integrin by appropriate ECM proteins causes conformational changes in integrin, which in turn initiate integrin activation and clustering to focal adhesion [8]. Focal adhesion kinase (FAK) aggregates to focal adhesion complex and FAK autophosphorylates [9], eventually resulting in the recruitment of other intracellular signaling proteins to integrin adhesion; this recruitment elicits various downstream signaling pathways to regulate cell survival, proliferation, migration, differentiation, cell shape, and contractility [8, 10]. β1 integrin is the predominant integrin expressed in the endothelial lining of CNS vessels [4, 11, 12]. Several β1-containing integrins are detected in the endothelial cells of the CNS vasculatures, including α1β1, α2β1, α3β1, α5β1, α6β1, and αvβ1 [13–15]. In healthy situations, β1 integrin–ECM engagement induces integrin signaling cascades that modulate the expression of the tight junction proteins and maintain the endothelial cell integrity of the CNS microvessels [4, 16]. However, under the pathological condition, ECM composition changes and β1 integrin–ECM interaction increases; thus, endothelial integrity is disrupted [13, 17].

Fibronectin (FN), the ligand of α5β1 integrin, is a heterodimeric ECM protein synthesized and secreted by several cell types in the CNS, including endothelial cells, pericytes, and astrocytes [15, 18]. FN expression is limited by its restriction to the subendothelial space of microvessels in the CNS [19–21]. Abnormal synthesis and excessive deposition of FN into the ECM is observed in the spinal cord after trauma or chronic inflammation in the peripheral nervous system or CNS [12, 22–25]. Our previous study demonstrated that increased FN accumulation in carotid arteries promotes leukocyte extravasation from the circulation into the vascular wall [26]. Studies have revealed that excessive FN deposition weans cell–cell adhesion in fibroblasts by increasing cell contractility in a β1-integrin-dependent manner [27, 28], suggesting that enhanced FN deposition in the pathological spinal cord compromises BSCB function through ligation to α5β1 integrin and elicits the downstream signaling cascades, leading to increased actomyosin interaction.

As reported previously [26], pUR4, a recombinant peptide derived from F1 adhesin [29], is an effective inhibitor of FN deposition in the vascular wall. In the present study, we used an in vitro model of an endothelial monolayer treated with tumor necrosis factor (TNF)-α to mimic a leaky BSCB in the spinal cord, and the effects of pUR4 on endothelial permeability were determined. Our study demonstrated that intact ECM FN is crucial for maintaining endothelial monolayer integrity and that excessive TNF-α-induced FN deposition enhances endothelial permeability by modulating β1 integrin–mediated stress fiber formation, actomyosin interaction, and paracellular gap formation. Furthermore, administration of pUR4 abrogates FN deposition and inhibits TNF-α-induced endothelial hyperpermeability. Thus, pUR4, which can decouple FN and β1 integrin, can be used as a novel therapeutic agent for treating endothelial dysfunction.

## Methods

### Materials

Scrambled peptides and pUR4 were custom synthesized by Kelowna International Scientific Inc. (Taipei, Taiwan; pUR4 sequence: GSKDQSPLAGESGETEYITEVYGNQQNPVDIDKKLPNETGFSGNMVETEDTKLN; scrambled peptide sequence: QGQTGPVNSKVKIDNYELESNPEKIEANDLQVEGTTTYESKFMGDLTGSGNPED). For in vitro assays, TNF-α was procured from Roche (Basel, Switzerland).

### Cell culture

Brain-derived endothelial (bEND.3) cells were provided by the Bioresource Collection and Research Center (Hsinchu, Taiwan). These cells were grown in Dulbecco’s Modified Eagle’s Medium supplemented with 10% fetal bovine serum. Human cerebral microvascular endothelial (hCMEC/D3) cells were purchased from Merck Millipore (Burlington, MA, USA) and maintained in EndoGRO-MV Complete Media Kit (Merck Millipore) supplemented with 1 ng/mL fibroblast growth factor (FGF)-2 (Merck Millipore). All cells were cultured under standard conditions (i.e., 5% CO_2_ in air in a humidified environment at 37 °C) for 7 days until the cell–cell junctions had been well established before being subjected to subsequent experiments.

### Evaluation of ECM FN assembled by bEND.3 cells

To evaluate ECM FN deposited by bEND.3 cells, the procedure of removing cells from FN assembled outside the cells was modified from previously reported protocols [30, 31]. In brief, cells were extracted using lysis buffer (20 mN Na_2_HPO4, pH 9.6, 1% Nonidet P-40) at room temperature for 10 min and then underwent several gentle washes with phosphate-buffered saline (PBS). ECM FN is largely preserved after this extraction process [30, 31]. The assembled ECM FN in the present study was then subjected to immunoblotting.

### Measurement of transendothelial electrical resistance

This study cultured bEND.3 cells (8.5 × 10^4^ cells) and hCMEC/D3 cells (8.5 × 10^4^ cells) on Transwell inserts (pore size = 0.4 μm; Merck Millipore) on a 24-well plate for 7 days until confluent. Cells were preincubated with 1000 nM pUR4, 1000 nM scrambled peptide, or PBS for 16 h and then underwent 20 ng/mL TNF-α or PBS treatment for 24 h. Transendothelial electrical resistance (TEER) was measured using an endothelial voltohmmeter with chopstick electrodes (Merck Millipore). During measurement, one electrode each was placed in the luminal and abluminal media. Resistance across the cell monolayer was measured (in ohm*cm^2^).

### Transendothelial permeability assays

This study grew bEND.3 cells (8.5 × 10^4^ cells) on Transwell inserts (pore size = 0.4 μm) on a 24-well plate for 7 days until confluent. The cells were preincubated with 1000 nM pUR4, 1000 nM scrambled peptide, or PBS for 16 h and then underwent 20 ng/mL TNF-α or PBS treatment for 24 h. Fluorescein isothiocyanate (FITC)–dextran (final concentration: 1 mg/mL, molecular weight: 40 kDa; Sigma-Aldrich, St. Louis, MO, USA) or sodium fluorescein (final concentration: 10 μg/mL, molecular weight: 376 Da; Sigma-Aldrich) was applied apically for 1 h, and the fluorescence intensity (FI) of the FITC–dextran and sodium fluorescein in the medium of the abluminal compartment were quantified using a SpectraMax M5 microplate reader (Molecular Device, Sunnyvale, CA, USA) set at 485 (excitation)/530 (emission) and 440 (excitation)/525 (emission) nm, respectively. Permeability was determined through FI_60min_/FI_0min_ and normalized to the PBS control [32].

### Immunofluorescence analysis and confocal microscopy

This study cultured bEND.3 cells (4.5 × 10^4^ cells) on cover glass for 7 days until confluent. After TNF-α treatment, the cells were washed in warm PBS and fixed in 1.75% paraformaldehyde for 15 min. The cells were subsequently incubated with PBS containing 0.1% Triton X-100 for 10 min (except for those subjected to immunofluorescence analysis for FN) and then blocked with 1% bovine serum albumin in PBS for 30 min at room temperature. After blocking, the cells were incubated with primary antibodies against FN (#AB2033, 1:200, Merck Millipore), ZO-1 (#61–7300 or #33–9100, 1:200, Thermo Fisher Invitrogen, Waltham, MA, USA), activated β1 integrin (clone HUTS-4, 1:100, Merck Millipore), β3 integrin (#13166, 1:200, Cell signaling, Denver, MA, USA), FAK (#3285, 1:200, Cell Signaling), and vascular endothelial (VE)-cadherin (#36–1900, 1:200, Thermo Fisher Invitrogen) overnight at 4 °C followed by Alexa Fluor 488– and 594–conjugated secondary antibodies (1:100, Thermo Fisher Invitrogen) for 1 h at room temperature. Some cells were coincubated with Alexa Fluor 488–conjugated phalloidin (1:200, Thermo Fisher Invitrogen). Confocal images were captured using a Zeiss LSM 780 confocal scanning microscope (Oberkochen, Germany), and the images were processed using Photoshop CS6 (Adobe, San Jose, CA, USA).

### Quantification of FI

Immunofluorescence analysis for activated β1 and β3 integrins was performed on bEND.3 cells after the various treatments. Digital color images were captured using a 100× objective and transformed into grayscale images. Three independent experiments were performed. In a representative experiment, three fields of view of equal size including 30–45 cells per treatment were randomly selected, and the mean FIs of the activated β1 and β3 integrin staining were analyzed using Image J (NIH, Bethesda, MD, USA).

### Assessment of cell morphology

To assess cell morphology, bEND.3 cells (4.5 × 10^4^ cells) were cultured on cover glass for 7 days until confluency was achieved. After pretreatment with pUR4 or scrambled peptide for 16 h, the cells were stimulated with TNF-α for 24 h. Immunofluorescence analysis for ZO-1 was performed to delineate the cell–cell junctions, and digital images were captured using a 20× objective. Three independent experiments were performed. In a representative experiment, 8–10 cells were randomly chosen for each image, and 7–10 images were used for each experimental group. Cell area and cell dimension (width:length ratio) were measured using Image-Pro Plus (Media Cybernetics, Rockville, MD, USA).

### Immunoblotting

The dorsal part of rat lumbar spinal cords, bEND.3 cells, assembled ECM FN derived from bEND.3 cells, and hCMEC/D3 cells were separately homogenized using a cold lysis buffer (50 mM Tris-HCl, pH 7.5, 150 mM NaCl, 1% NP-40, and 5 mM ethylenediaminetetraacetic acid) supplemented with protease and a phosphatase inhibitor cocktail (Sigma-Aldrich). Concentrated media and cell and tissue lysate were electrophoresed on a sodium dodecyl sulfate–polyacrylamide electrophoresis gel under reducing conditions and transferred to nitrocellulose membranes. The membranes were probed with antibodies against ZO-1 (1:1000, #61–7300, Thermo Fisher Invitrogen), occludin (1:1000, #71–1500, Thermo Fisher Invitrogen), claudin-5 (1:1000, 4C3C2, Thermo Fisher Invitrogen), VE-cadherin (#36–1900, 1:200, Thermo Fisher Invitrogen), phosphorylated myosin light chain (MLC) 9 (1:1000, 18HCLC, Thermo Fisher Invitrogen), FAK (1:1000, #3285, Cell signaling), phospho-FAK (Tyr397; 1:1000, D20B1, Cell signaling), phospho-FAK (Tyr576/577; 1:1000, #3281, Cell signaling), MLC9 (1:1000,PA5–43153, Thermo Fisher Invitrogen), FN (1:5000, F3648, Sigma-Aldrich), glyceraldehyde-3-phosphate dehydrogenase (GAPDH; 1:5000, G9545, Sigma-Aldrich), or α-tubulin (1:5000, B-5-1-2, Sigma-Aldrich) overnight at 4 °C followed by horseradish peroxidase–conjugated secondary antibodies (Thermo Fisher Invitrogen) for 1 h at room temperature. Quantitative analysis was performed through densitometry in Image J (NIH).

### Quantitative real-time polymerase chain reaction

Total RNA from cells was isolated using TRIzol (Thermo Fisher Invitrogen). Total RNA from each sample was reverse transcribed with a First Strand cDNA Synthesis Kit (GE Healthcare Life Sciences, Marlborough, MA, USA) according to the manufacturer’s instructions. Quantitative real-time polymerase chain reaction (PCR) was performed using multiple sets of quantitative PCR primers (mouse ZO-1 primers: forward 5′- CATCATTCGCCTTCATACAA-3′, reverse 5′- ACACAACCTCATCCTCATT-3′; mouse claudin-5 primers: forward 5′-GGCACCAGAATCAATTCC-3′, reverse 5′-CCATCCTACCAGACACAG-3′; mouse occludin primers: forward 5′-TATGGCGGATATACAGACC-3′, reverse 5′- ATTACTAAGGAAGCGATGAAG-3′) and a SensiFAST SYBR No-ROX Kit (Bioline, London, UK) on a CFX96 Real-Time PCR Detection System (Bio-Rad, Hercules, CA, USA). Quantitative real-time PCR was performed on TNF-α-treated bEND.3 cells incubated with pUR4 or scrambled peptide. Each quantitative real-time PCR experiment was performed at least three times, and the representative results were expressed as a fold change relative to the control using the standard 2^−ΔΔCt^ method [33], where Ct is the number of cycles required to reach the threshold for the target gene subtracted from the number of cycles required to reach the threshold for a control housekeeping gene (GAPDH in this study).

### Spinal nerve ligation injury

Eight-week-old male Sprague Dawley rats weighing 250–350 g were subjected to surgery. All surgical procedures were performed under anesthesia through 2% isoflurane inhalation. The L5 spinal nerve distal to the dorsal root ganglion on the right side was exposed and tightly ligated with a silk suture (5–0).

### Tissue collection and immunohistochemistry

Rats were anesthetized with an overdose of isoflurane and intracardially perfused with 10% formalin 7 days after surgery. The rats’ L5 lumbar spinal cords were dissected, cryoprotected with 30% (w/v) sucrose in PBS, and then cut into 30-μm-thick sections. Cryosections were incubated with polyclonal anti-FN antibody (#AB2033, 1:8000, Merck Millipore) overnight at 4 °C followed by biotinylated secondary antibodies for 1 h at room temperature. Sections were incubated with an avidin-biotin immunoperoxidase system (Vector Laboratories, Burlingame, CA, USA) for 30 min. A Liquid DAB Substrate Chromogen system (Dako, Carpinteria, CA, USA) was used for detection. To quantitate FN expression in the spinal cord, immunofluorescence analysis for FN was performed. Fluorescence images of the operated and contralateral sides of the L5 spinal cords with a 4× objective were captured. Three to six fields of view of equal size were selected from each side, and the positively stained area for FN was obtained using an automated programmed segmentation procedure in Image-Pro Plus (Media Cybernetics). The percentage of the positively stained area to the total traced area on the operated or contralateral sides was determined. Data from three mice were averaged. To confirm that FN was expressed in capillaries, spinal cord sections were incubated with antibodies against FN (Clone IST-9, 1:200, Abcam, Cambridge, UK) and collagen type IV (#ab6586, 1:100, Abcam) to identify the basement membrane of brain capillary [34, 35].

### Statistical analysis

After tests for normality and equal variance had been conducted, appropriate statistical analyses were performed. The type of analysis used is indicated in the legend of each figure. For in vivo experiments, the data are presented as means ± standard errors of means. For in vitro experiments, data are expressed as means ± standard deviations of representative experiments; at least three independent experiments were performed. Student’s *t* test, a one-way analysis of variance (ANOVA), or a two-way ANOVA followed by a post hoc test were conducted for data analysis in GraphPad Prism (GraphPad, San Diego, CA, USA). *P* > 0.05 was considered nonsignificant.

## Results

### Spinal nerve ligation increases FN deposition in microvessels in the spinal cord

**Fig. 1:**
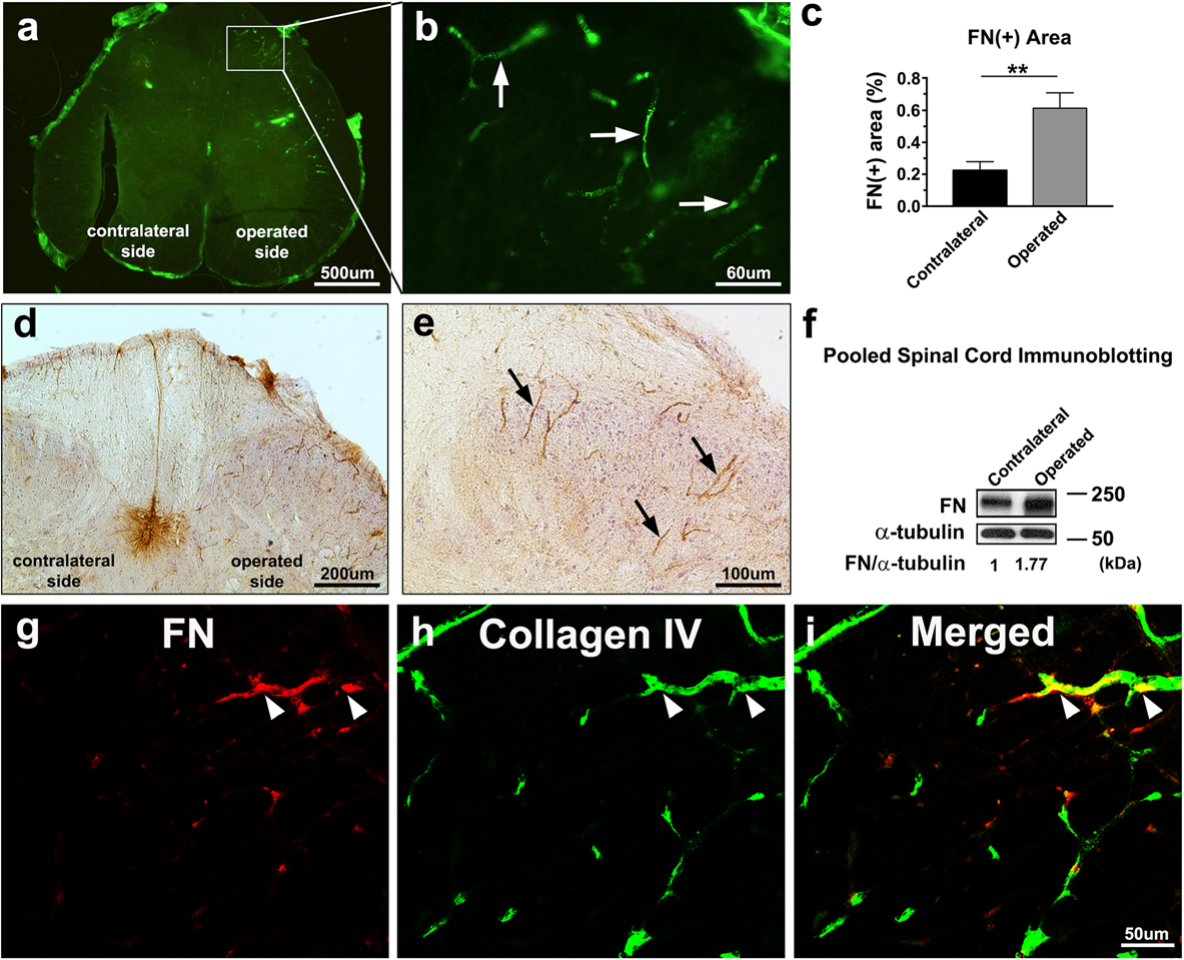
Spinal nerve ligation induces excessive FN deposition around microvessels in the spinal cord. The L5 spinal nerve distal to the dorsal root ganglion of rats was tightly ligated. **a** Representative photomicrograph depicting immunofluorescence analysis for FN on the operated and contralateral sides of the L5 segment of the spinal cord 7 days after surgery. **b** Regional magnification of the boxed area in (**a**); FN^+^ microvessel-like profiles are indicated by arrows. **c** Percentages of the FN^+^ area in the total area on the operated and contralateral sides of the L5 segment of the spinal cord were analyzed (*n* = 3). Data are presented as means ± standard errors of means. **P < 0.01, Student’s *t* test. **d** and **e** Representative images at low (**d**) and high (**e**) magnification showing immunocytochemistry of FN in the L5 dorsal part of the spinal cord. Arrows indicate FN^+^-microvessel-like profiles on the operated side of the spinal cord. **f** Immunoblotting for FN expression in the pooled L5 dorsal spinal cord on the operated and contralateral sides in five male Sprague Dawley rats. Equal protein loading was confirmed with α-tubulin. Quantification of immunoblotting of FN normalized to α-tubulin in tissues is shown. **g**–**i** Confocal microscopic images of FN^+^-microvessel-like profiles (red; g) and collagen IV^+^ capillaries (green; **h**) in the L5 dorsal part of the spinal cord; merged images (**i**) showing the colocalization of FN and collagen IV (yellow) in the capillaries are indicated with arrowheads

BSCB leakage is associated with several pathological conditions in the spinal cord, including multiple sclerosis, spinal cord injury, and peripheral nerve injury [36]. After peripheral nerve injury, FN expression is upregulated in the spinal cord [24, 37]. To ascertain the reproducibility of the data in our laboratory, the rats were subjected to L5 spinal nerve ligation. FN expression was evaluated in the L5 segment of spinal cords 7 days after ligation through immunofluorescence analysis. As shown in Fig. 1a and b, FN deposition was notably increased in the microvessel-like profiles on the operated side of the L5 spinal cord. Quantitative analysis revealed a significantly greater FN^+^ area on the operated side than on the contralateral side (Fig. 1c). Immunohistochemistry demonstrated FN^+^-microvessel-like structures mostly distributed in the dorsal spinal cord on the operated side (Fig. 1d and e). Immunoblotting of FN exhibited similar upregulation in protein levels in the pooled L5 dorsal spinal cord on the operated side in five male Sprague Dawley rats (Fig. 1f). To confirm that excessive FN was accumulated in the microvessels, coimmunostaining of FN and collagen type IV, which can be used to localize CNS capillaries [34, 35], was performed. As shown in Fig. 1g, h, and i, the expression of FN and collagen type IV was colocalized in the string-like microvessels, indicating that enhanced FN was accumulated in the basement membrane of the microvessels in the spinal cord. In our previous study, enhanced FN accumulated in the vascular wall potentially disrupted the endothelial barrier function of blood vessels [26]. Therefore, we speculated whether excessive FN deposition around the microvessels of the spinal cord impairs BSCB integrity after peripheral nerve injury.

### TNF-α-induced FN deposition is blocked by pUR4 blocks in bEND.3 cells

**Fig. 2:**
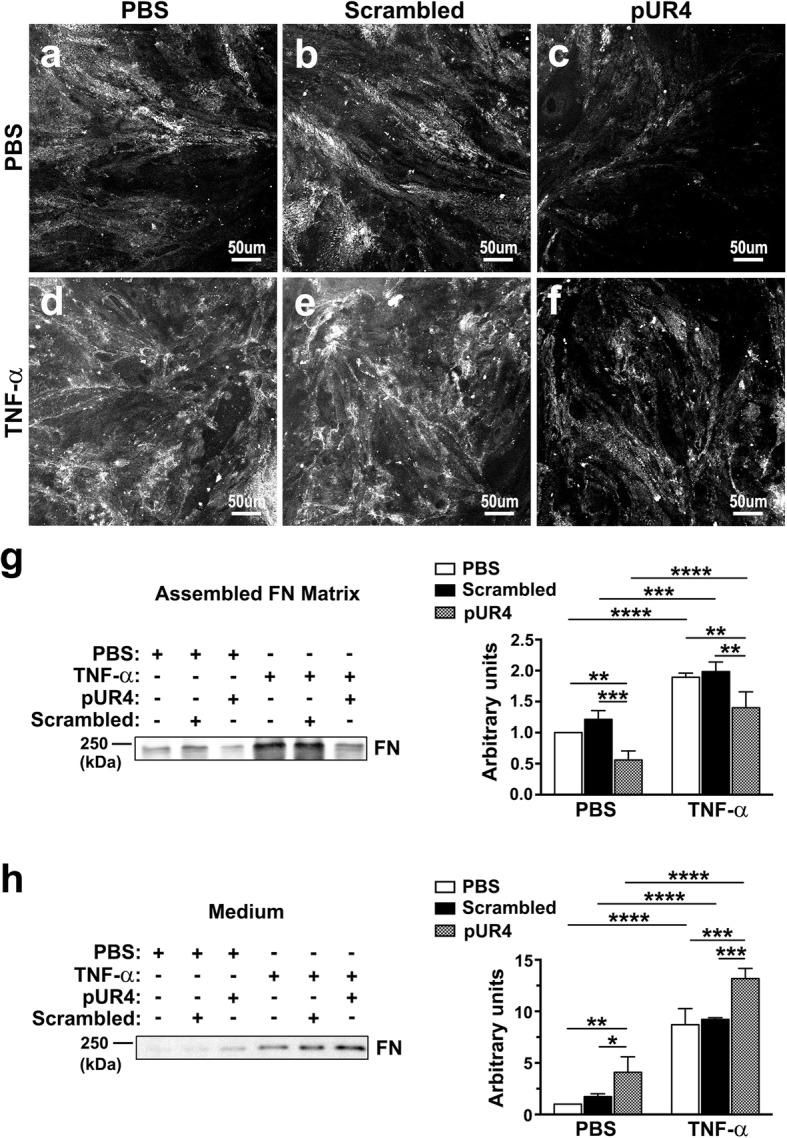
TNF-α-induced FN deposition in bEND.3 cells is inhibited by pUR4. After being incubated with 1000 nM scrambled peptide, 1000 nM pUR4, or PBS for 16 h, bEND.3 cells underwent treatment with TNF-α (20 ng/mL) or PBS for 24 h. **a**–**f** Immunofluorescence analysis for FN was performed on nonpermeabilized cells to evaluate FN fibrils accumulated outside bEND.3 cells. **g**–**h** To assess the extracellular matrix FN deposited by bEND.3 cells after the various treatments, cellular components were extracted using the lysis buffer, as described in the Methods section, and the assembled ECM FN by bEND.3 cells (**g**) and FN in the corresponding conditioned medium (**h**) were immunoblotted. Quantification of protein band intensity was determined (*n* = 3). Data are presented as means ± standard deviations. *P < 0.05, **P < 0.01, ***P < 0.001, and ****P < 0.0001, two-way ANOVA followed by Tukey’s multiple comparison test and Sidak’s multiple comparison test

To elucidate ECM FN regulation in the BSCB, we used an in vitro model of an endothelial monolayer with TNF-α treatment to mimic a leaky BSCB in vivo. The immortalized mouse brain endothelial cell line bEND.3 is strongly characterized by its tight paracellular barrier and is a popular cell line for BBB research [38–40]. TNF-α-induced endothelial hyperpermeability is a critical contributor to CNS inflammation [41, 42]. Moreover, L5 spinal nerve ligation such as that performed in this study can increase TNF-α expression in the spinal cord [43]. Therefore, we inferred that TNF-α is an appropriate cytokine to induce FN deposition and a leaky endothelium of the bEND.3 monolayer. First, we assessed the efficacy of pUR4 on the inhibition of FN accumulation in bEND.3 cells. The FN inhibitor pUR4 is a 49-mer peptide derived from *Streptococcus pyogenes* adhesion F1 that binds to the N-terminal modules of FN and inhibits soluble FN polymerization and deposition into the matrices of endothelial cells and many other cell types, including vascular smooth muscle cells, fibroblasts, and hepatocytes [26, 29, 44–46]. To optimize the pUR4 dose for inhibiting FN deposition into the ECM, various doses of pUR4 were applied to bEND.3 cells. A dose of 1000 nM of pUR4 significantly reduced extracellular FN fibril formation compared with the PBS-treated cells, as demonstrated through immunofluorescence analysis (Fig. 2a and c) and immunoblotting for the ECM FN assembled by bEND.3 cells (Fig. 2g). However, 3000 nM pUR4 almost depleted all ECM FN around the cells (Additional file 1: Figure S1b), and adhesion of the bEND.3 cells was considerably impaired (Additional file 1: Figure S2b). Notably, 1000- and 3000-nM doses of the scrambled peptide exerted no apparently harmful effects on FN deposition by bEND.3 cells (Fig. 2b and g, Additional file 1: Figure S1a) or cell adhesion (Additional file 1: Figure S2a). Therefore, we selected 1000 nM pUR4 for subsequent experiments. We next evaluated whether FN deposition in bEND.3 cells is induced by TNF-α. After stimulation by TNF-α for 24 h, FN substantially accumulated in the ECM of bEND.3 cells compared with the unstimulated cells treated with PBS, as shown through immunofluorescence analysis and immunoblotting (Fig. 2a, d, and g). Under TNF-α treatment, the addition of pUR4 to cultured bEND.3 cells significantly inhibited the deposition of TNF-α-induced FN fibrils in the ECM compared with the PBS control and scrambled peptide treatment, as shown through immunofluorescence analysis (Fig. 2d, e, and f) and immunoblotting (Fig. 2g). Our previous study demonstrated that pUR4 only inhibits FN deposition into the ECM without altering mRNA expression of FN [26]. Therefore, when preventing FN accumulation in the matrix in the presence or absence of TNF-α stimulation, pUR4 released more unincorporated FN into the conditioned media compared with the PBS control and scrambled peptide treatment (Fig. 2h). Taken together, these results confirm that pUR4 can inhibit TNF-α-induced FN assembly in bEND.3 cells.

### TNF-α-induced endothelial hyperpermeability is ameliorated by pUR4

**Fig. 3:**
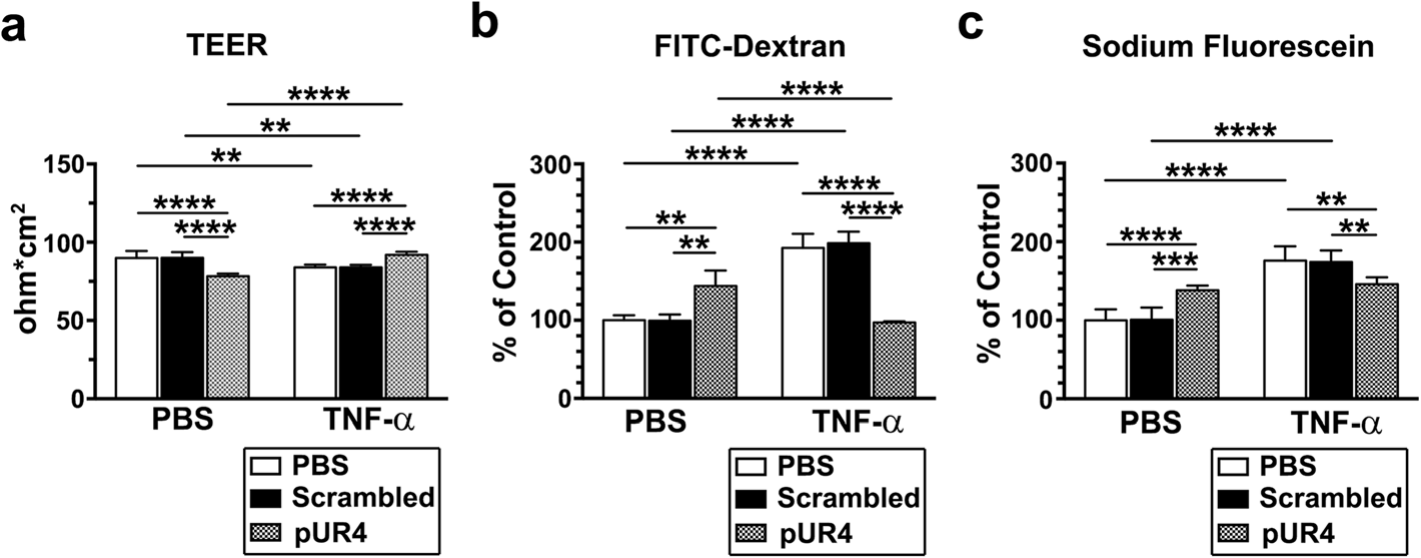
TNF-α-induced monolayer hyperpermeability was prevented by pUR4. After being grown to confluence on Transwell inserts, bEND.3 cells were preincubated with 1000 nM scrambled peptide, 1000 nM pUR4, or PBS for 16 h and then underwent treatment with TNF-α (20 ng/mL) or PBS control for 24 h. **a** TEER assay was performed 24 h after TNF-α or PBS treatment to determine bEND.3 monolayer integrity (*n* = 3 for each experimental group). Data are presented as means ± standard deviations. **P < 0.01 and ****P < 0.0001, two-way ANOVA followed by Tukey’s multiple comparison test and Sidak’s multiple comparison test. **b** and **c** 40-kDa FITC–dextran (final concentration = 1 mg/mL; **b**) and sodium fluorescein (final concentration = 10 ng/mL; **c**) were loaded into the upper chamber for 1 h after 24-h treatment with TNF-α or PBS control, and the FI of the medium in the lower chamber was measured. Permeability was determined through FI_60min_/FI_0min_ and normalized to the PBS control (n = 3). Data are presented as means ± standard deviations. **P < 0.01, ***P < 0.001, and ****P < 0.0001, two-way ANOVA followed by Tukey’s multiple comparison test and Sidak’s multiple comparison test

We next sought to determine whether pUR4 prevents TNF-α-induced barrier dysfunction in the inflamed endothelial monolayer through TEER measurement. After being grown on Transwell inserts, bEND.3 cells were preincubated with PBS (control), scrambled peptide, or pUR4 for 16 h and then stimulated with TNF-α for 24 h or left unstimulated. Treatment of cells with 1000 nM pUR4 alone, which can inhibit ECM FN deposition (Fig. 2c), significantly reduced endothelial TEER compared with treatment with scrambled peptide no stimulation (Fig. 3a), indicating that intact ECM FN is critical for maintaining endothelial integrity. After 24 h of TNF-α stimulation, TEER in the bEND.3 monolayer preincubated with PBS control or scrambled peptide was significantly attenuated compared with that in the cells left unstimulated by PBS (Fig. 3a). By contrast, pretreatment of cells with pUR4 prevented a TNF-α-induced TEER reduction (Fig. 3a). We also evaluated the barrier function of bEND.3 cells by using a permeability assay. The bEND.3 cells treated with pUR4 alone exhibited higher paracellular permeability to FITC–dextran (Fig. 3b) and sodium fluorescein (Fig. 3c) than did those treated with PBS control or scrambled peptide alone. After TNF-α stimulation, the addition of pUR4 prevented an increase in endothelial permeability for FITC–dextran and sodium fluorescein induced by TNF-α (Fig. 3b and c). To confirm that the effect of pUR4 on the reduction of TNF-α-induced endothelial hyperpermeability is common for other endothelial monolayers, hCMEC/D3 cells, a well-characterized in vitro model of the human BBB [47], were subjected to the TEER assay. As shown in Additional file 1: Figure S3a, pUR4 exerted the same effects in TNF-α-stimulated and -unstimulated hCMEC/D3 cells as in the bEND.3 cells (Fig. 3a). Taken together, these results indicate that pUR4 efficiently restores the TNF-α-compromised endothelial barrier function.

### TNF-α-induced endothelial hyperpermeability is not attenuated by pUR4 altering the expression of tight and adherens junction proteins

**Fig. 4:**
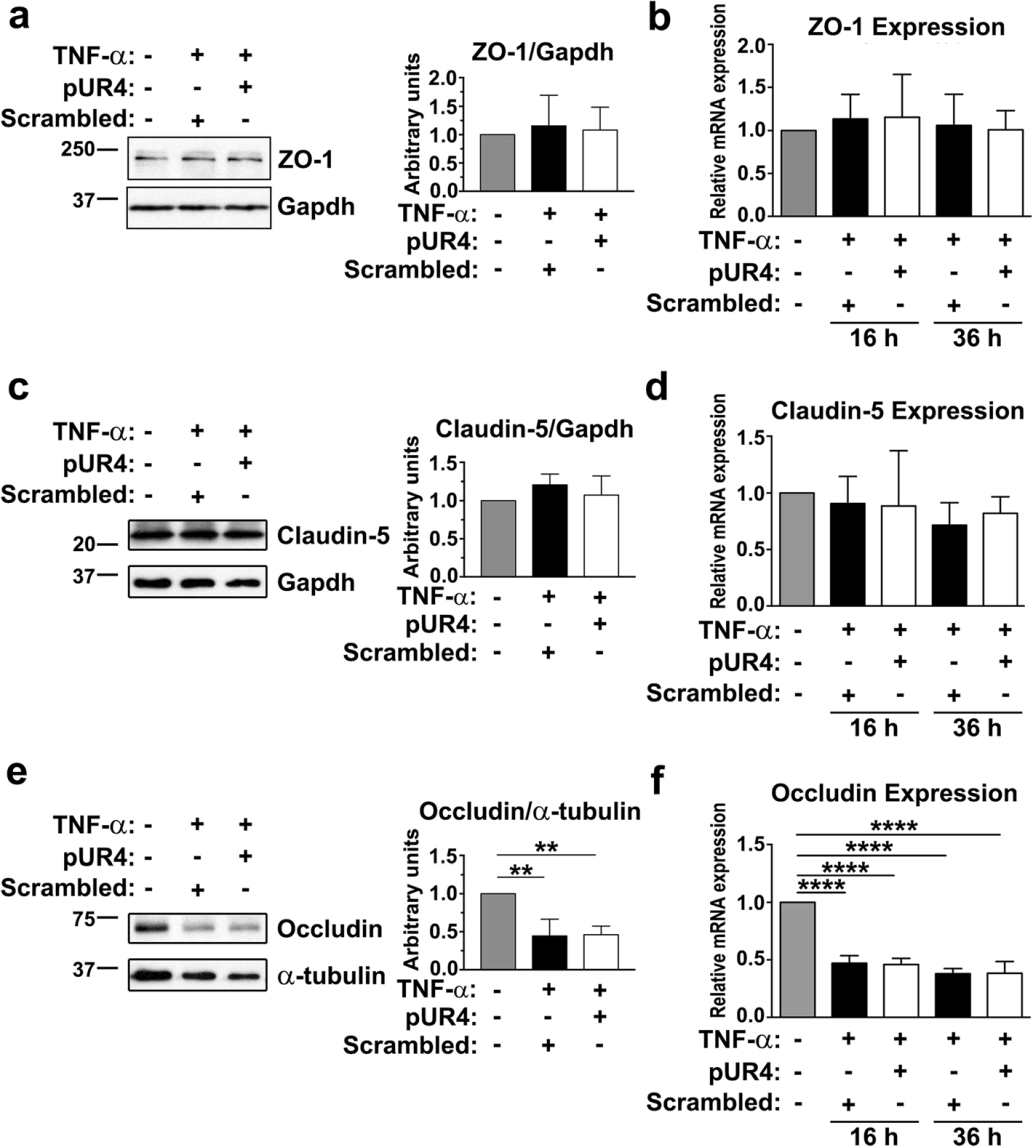
Tight junction protein expression is not altered by pUR4. After being pretreated with 1000 nM scrambled peptide or 1000 nM pUR4 for 16 h, bEND.3 cells were stimulated with 20 ng/mL TNF-α for the indicated durations. The protein expression of ZO-1 (**a**), claudin-5 (**c**), and occludin (**e**) in bEND.3 cells 24 h after TNF-α treatment was evaluated through immunoblotting; the quantitative analysis results for of ZO-1, claudin-5, and occludin are normalized to GAPDH or α-tubulin (n = 3). ZO-1 (**b**), claudin-5 (**d**), and occludin (**f**) transcript expression in bEND.3 cells treated with TNF-α for 16 and 36 h was evaluated using quantitative real-time PCR (n = 3). Data are presented as means ± standard deviations. **P < 0.01 and ****P < 0.0001, one-way ANOVA followed by Tukey’s multiple comparison test

We next determined whether pUR4-regulated attenuation of endothelial barrier disruption induced by TNF-α was caused by the alteration of tight junction proteins; thus, expression of the tight junction cytoplasmic protein ZO-1 and the transmembrane proteins claudin-5 and occludin was assessed through immunoblotting and quantitative real-time PCR after TNF-α treatment. TNF-α exposure induced no significant changes in protein or mRNA levels of ZO-1 or claudin-5 in bEND.3 cells (Fig. 4a–d). Similar results were also noted for hCMEC/D3 cells (Additional file 1: Figure S3b and c). By contrast, the protein expression of occludin in bEND.3 cells decreased at 24 h after TNF-α exposure (Fig. 4e). Similarly, 16 and 36 h after TNF-α treatment, the occludin mRNA level was significantly attenuated (Fig. 4f). These results are consistent with those reported previously in that TNF-α resulted in reduced expression of occludin in bEND.3 cells but did not affect the protein level of ZO-1 or claudin-5 [48]. Preincubation of pUR4 in TNF-α-treated bEND.3 cells did not elicit any significant change in the protein or mRNA level of ZO-1 (Fig. 4a and b), claudin-5 (Fig. 4c and d), or occludin (Fig. 4e and f) compared with the scrambled peptide pretreatment in TNF-α-treated bEND.3 cells. Adherens junctions have a tight junction supportive role in forming the barrier function in cerebral endothelial cells [49]. Therefore, we determined the expression of VE-cadherin, a component of adherens junctions, in bEND.3 cells through immunoblotting (Additional file 1: Figure S4a and b). TNF-α stimulation substantially attenuated the expression of VE-cadherin, and the addition of pUR4 did not significantly restore the protein levels of VE-cadherin. These results collectively demonstrate that pUR4 does not modulate TNF-α-induced endothelial leakage by upregulating the expression of tight and adherens junction proteins.

### Stress fiber formation and actomyosin interaction is reduced by pUR4 in the TNF-α-treated endothelial monolayer

**Fig. 5:**
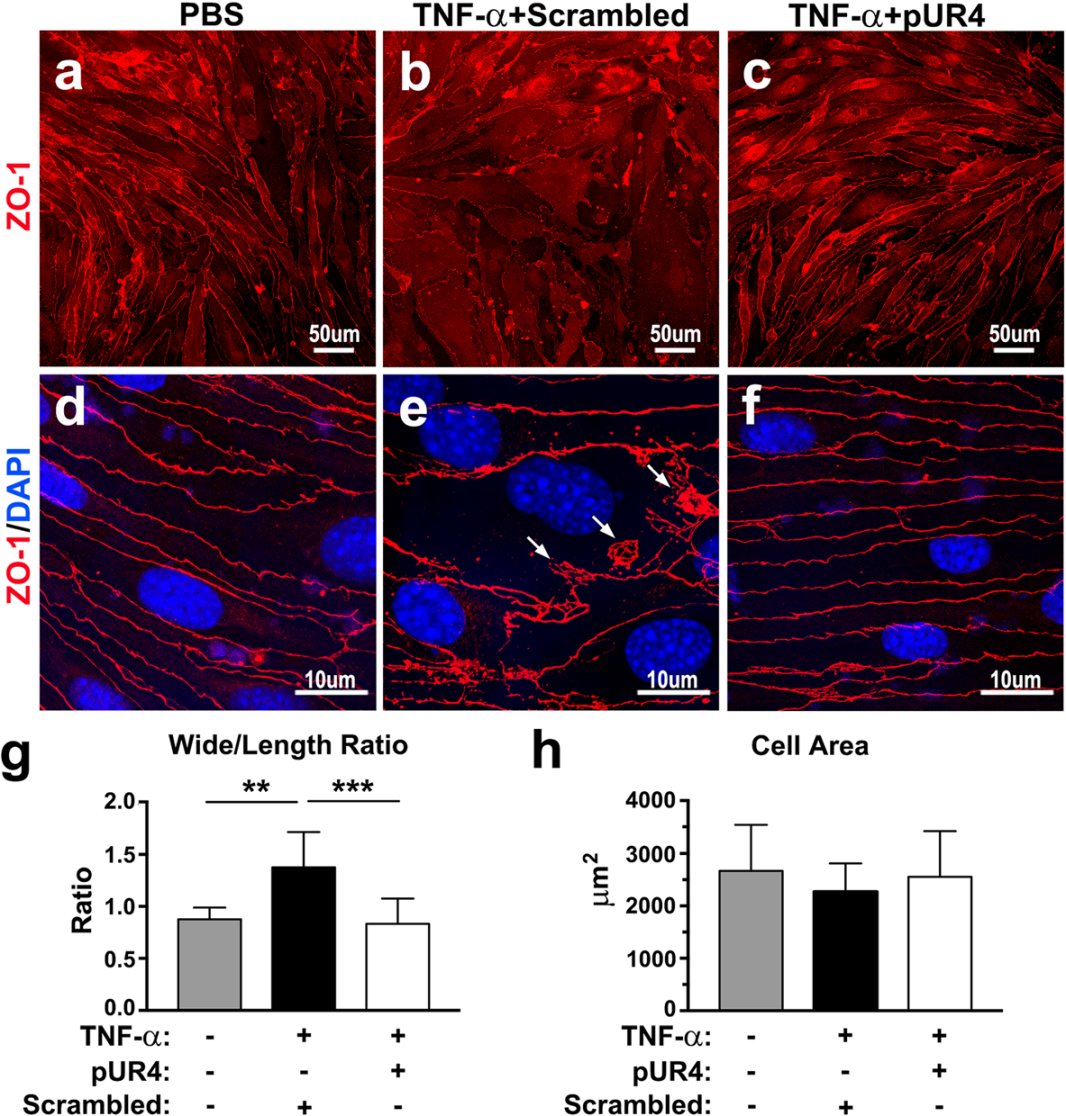
TNF-α-induced alteration of endothelial morphology is prevented by pUR4. After being preincubated with 1000 nM scrambled peptide or 1000 nM pUR4 for 16 h, bEND.3 cells were treated with TNF-α for 24 h. PBS-treated control and TNF-α-treated bEND.3 cells were immunostained with anti-ZO-1 antibody to depict the cell boundaries. Three independent experiments were performed, and each experiment was repeated with similar results. Representative photomicrographs at low (**a**)–(**c**) and high (**d**)–(**f**) magnification show the endothelial cell shapes. Paracellular gaps are marked by arrows. Cell width:length ratio (width/length ratio) (**g**) and quantitative analysis of the cell area delineated by ZO-1 (**h**) derived from a single representative experiment are shown (*n* = 7–9). Data are expressed as means ± standard deviations. **P < 0.01 and ***P < 0.001, one-way ANOVA followed by Tukey’s multiple comparison test

Exposure of endothelial cells to TNF-α can alter the cell morphology because of the formation of actin stress fiber [32, 50]. To correlate the inhibitory effects of pUR4 on TNF-α-induced endothelial disruption with changes in endothelial morphology, PBS control and TNF-α-treated bEND.3 monolayers were immunostained for ZO-1 to delineate the cell–cell boundaries. Confocal microscopy revealed unstimulated individual bEND.3 cells treated with PBS that exhibited stretched morphologies with relatively low width:length ratios (Fig. 5a and g); immunostaining for ZO-1 indicated a well-defined, contiguous border between adjoining cells (Fig. 5d). By contrast, the stretched morphologies of individual TNF-α-treated bEND.3 cells were impaired, and the cells were rounded with higher width:length ratios compared with the unstimulated cells (Fig. 5b and g). Moreover, bEND.3 cells treated with TNF-α demonstrated that ZO-1 was disorganized at the cell border, and the formation of paracellular gaps was observed (see the arrows in Fig. 5e). After the addition of pUR4, a stretched morphology of the bEND.3 cells similar to that of the unstimulated cells was maintained (Fig. 5c and g), and immunofluorescence indicated continuous distribution of ZO-1 upon cell–cell contact (Fig. 5f). Moreover, measurement of the individual cell areas in Image-Pro Plus revealed no significant differences between the unstimulated cells and TNF-α-treated cells in the presence or absence of pUR4 (Fig. 5h). We also delineated the cell–cell boundaries by immunolabeling VE-cadherin. Reduced staining intensity with a discontinuous VE-cadherin^+^ border and paracellular gaps was evident after TNF-α treatment (Additional file 1: Figure S4d). After the addition of pUR4, the stretched cell morphology evidenced by the immunofluorescence analysis for VE-cadherin was maintained (Additional file 1: Figure S4e).

**Fig. 6:**
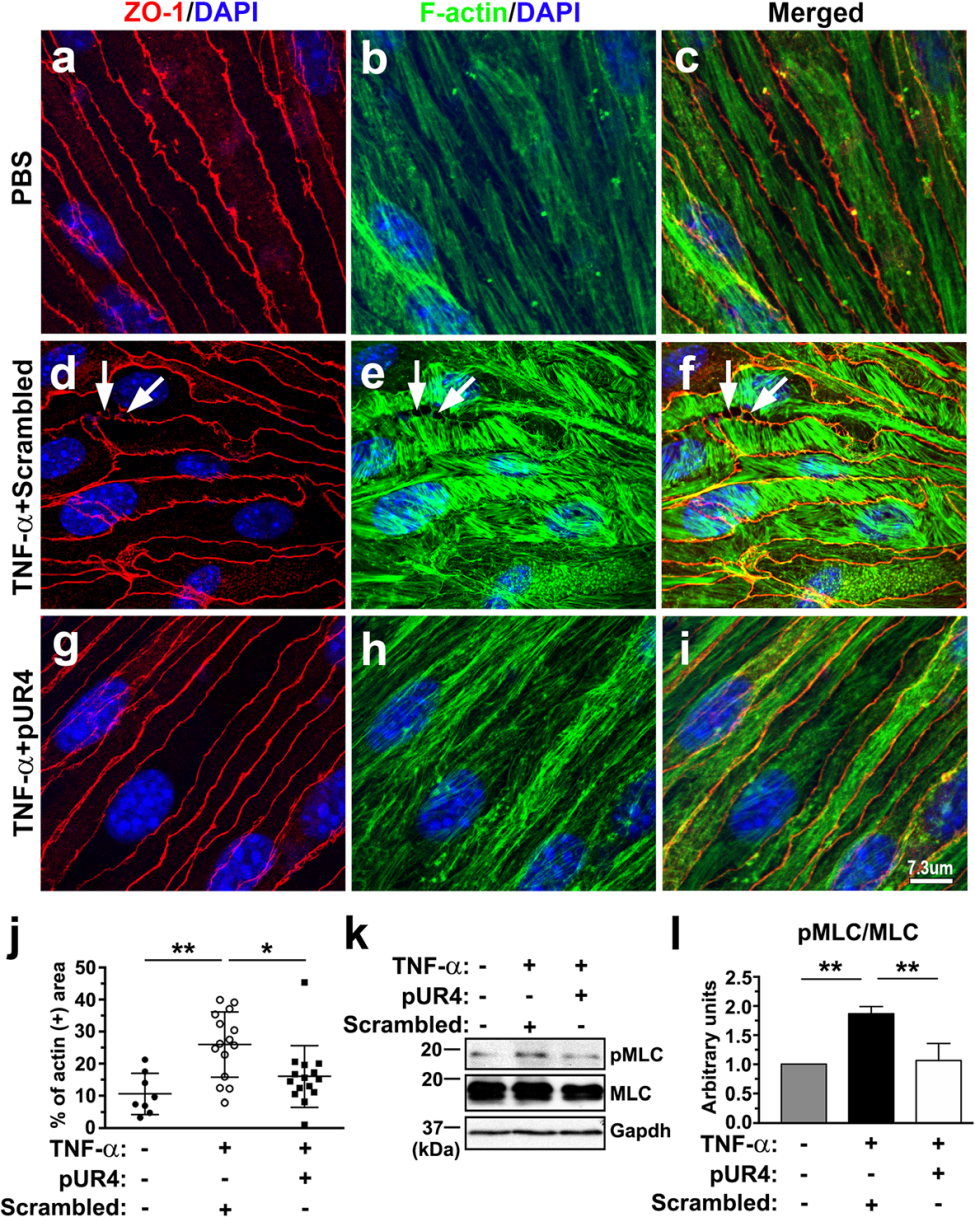
TNF-α-induced stress fiber formation and MLC phosphorylation are attenuated by pUR4. After being pretreated with 1000 nM scrambled peptide or 1000 nM pUR4 for 16 h, bEND.3 cells were stimulated with 20 ng/mL TNF-α for 24 and 6 h. **a**–**i** Cytoskeletal remodeling in response to TNF-α treatment for 24 h was analyzed through immunofluorescence analysis for ZO-1 (red) and phalloidin staining for F-actin (green) in PBS control and TNF-α-treated bEND.3 cells. Arrows indicate paracellular gaps. **j** Quantitative analysis of the F-actin^+^ area in the total area in PBS control and TNF-α-treated bEND.3 cells with scrambled peptide or pUR4 preincubation (n = 3). Data are expressed as means ± standard deviations. *P < 0.05 and **P < 0.01 by one-way ANOVA followed by Tukey’s multiple comparison test. **k** and **l** After exposure to TNF-α for 6 h, MLC phosphorylation was examined by immunoblotting with phospho-MLC (T18/S19)-specific antibody (pMLC), and total MLC (MLC) antibody on the same membrane after stripping (k). Quantification of immunoblotting of pMLC normalized to MLC in the bEND.3 cells (l; n = 3). Data are represented as means ± standard deviations. **P < 0.01, one-way ANOVA followed by Tukey’s multiple comparison test

Because the actin cytoskeleton is critical for maintaining cell morphology, we subsequently attempted to determine whether pUR4 prevents TNF-α-induced actin rearrangement. After treatment with TNF-α for 24 h, numerous thick actin stress fibers traversed the scrambled peptide–pretreated bEND.3 cells over the nucleus, as observed through phalloidin staining (Fig. 6e and f). Compared with unstimulated cells treated with PBS, TNF-α-treated bEND.3 cells exhibited larger F-actin^+^ areas (Fig. 6j). The bundles of stress fibers perpendicular to the cell–cell contact delineated through the immunostaining of ZO-1 were associated with the appearance of paracellular gaps (Fig. 6d–f). By contrast, the bEND.3 cells preincubated with pUR4 and then stimulated with TNF-α for 24 h reduced F-actin assembly, as demonstrated by the smaller F-actin^+^ area in Fig. 6j, and maintained the actin bundles parallel to the cell–cell junction (Fig. 6g–i) similar to those in the unstimulated cells (Fig. 6a–c). Evidence has indicated that TNF-α induces endothelial dysfunction by activating Rho GTPase and MLC kinase, leading to the phosphorylation of Ser 19 in MLCs [48, 50]; this leads to the assembly of stress fiber and actomyosin contractility [51–53]. Therefore, we next sought to determine whether pUR4 decreased actomyosin interaction by evaluating the phosphorylated state of MLCs. As shown in Fig. 6k and l, pUR4 treatment significantly attenuated TNF-α-induced MLC phosphorylation in bEND.3 cells. Taken together, these data indicate that pUR4 attenuated TNF-α-induced paracellular gap formation by minimizing stress fiber formation and actomyosin interaction.

### TNF-α-induced activation of β1 integrin and downstream signaling are reduced by pUR4 in endothelial cells

**Fig. 7:**
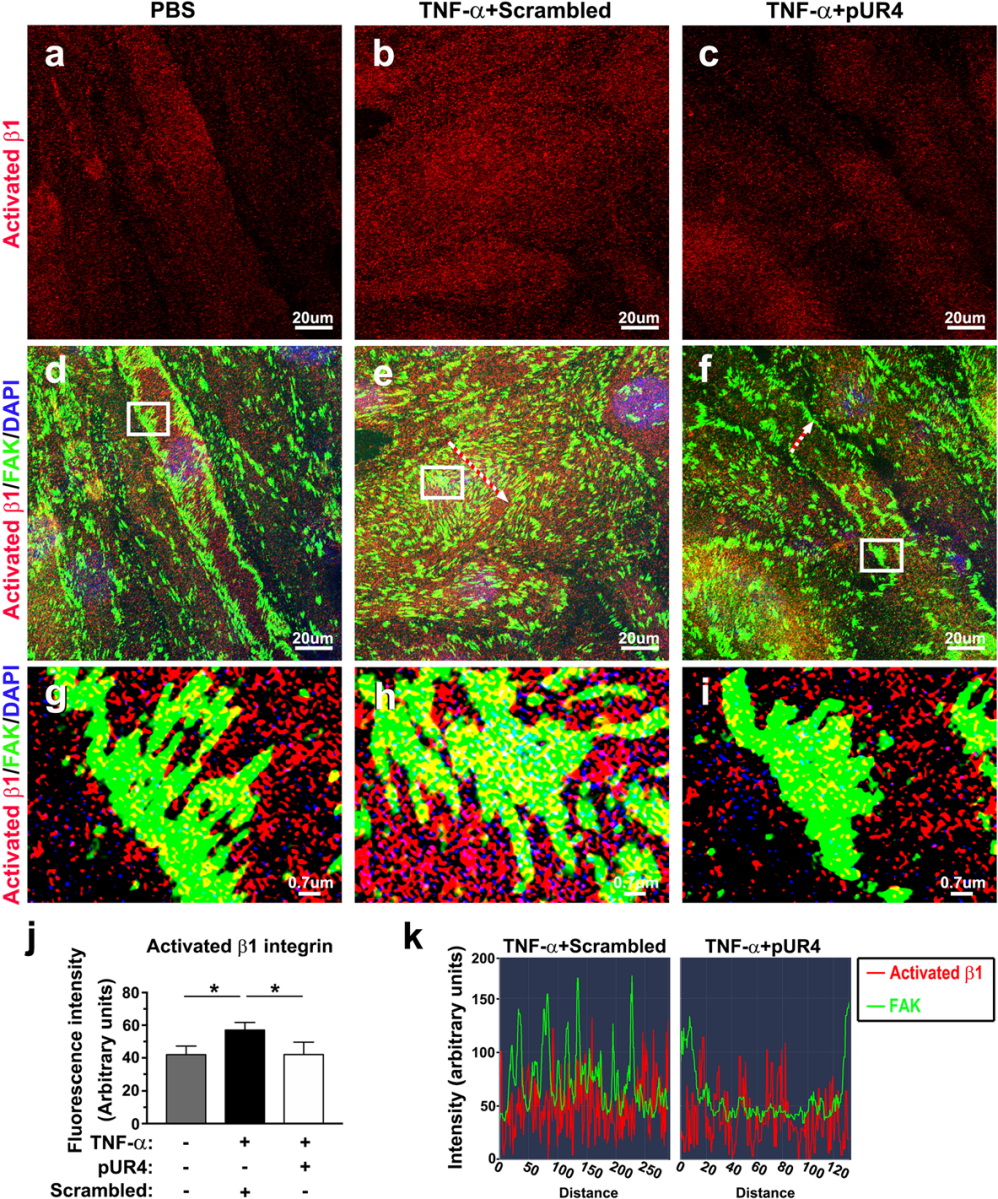
TNF-α-induced β1 integrin activation and clustering to focal adhesions (FAs) are diminished by pUR4. After being pretreated with 1000 nM scrambled peptide or 1000 nM pUR4 for 16 h, bEND.3 cells were stimulated with TNF-α (20 ng/mL) for 24 h. PBS-treated control and TNF-α-treated bEND.3 cells were costained with antibodies to the activated state of β1 integrin (red) and FAK (green). Three independent experiments were performed, and each experiment was repeated with similar results. **a**–**c** Representative immunofluorescence images showing activated β1 integrin in control and bEND.3 cells treated with the scrambled peptide and pUR4.**d**–**f** The merged images show colocalization (yellow) of activated state of β1 integrin (red) and FAK (green). **g**, **h**, and **i** Reginal enlargement of the FAs in the boxed area in (**d**), (**e**), and (**f**), respectively. (**j**) Quantification of FI of activated β1 integrin in PBS control and bEND.3 cells treated with the scrambled peptide and pUR4 was derived from a representative experiment (n = 3). Data are represented as means ± standard deviations. *P < 0.05, one-way ANOVA followed by Tukey’s multiple comparison test. (**k**) Line scan graphs showing the immunofluorescence intensity along the freely positioned arrows in bEND.3 cells treated with scrambled peptide (**e**) and pUR4 (**f**)

ECM–β1 integrin coupling or integrin activation by its ligands, including FN, regulates endothelial permeability by increasing actin stress fiber formation and cell contractility [54–56]. Endothelial cells of the CNS express several β1 integrins; however, α5β1 is the only β1 integrin receptor for FN. We next determined whether the inhibitory effect of pUR4 on TNF-α-induced stress fiber formation and actomyosin interaction was attributable to decreased integrin engagement. We evaluated the activation state of β1 integrin through immunofluorescence 24 h after the treatment of TNF-α in bEND.3 cells; cell–ECM adhesions were labeled by costaining the cells with anti-FAK antibody. After TNF-α stimulation, the FI of activated β1 integrin in bEND.3 cells was substantially increased compared with that in unstimulated cells (Fig. 7a, b, and j). Enhanced cell–ECM adhesion, as evidenced by robust FAK staining and accompanied by increased colocalization of activated β1 integrin and FAK, was noted after TNF-α treatment (Fig. 7d, e, g, and h), indicating β1 integrin clustering in focal adhesions. Inhibition of FN accumulation with pUR4 significantly diminished the activation of β1 integrin induced by TNF-α (Fig. 7b, c, and j). Moreover, pUR4 attenuated TNF-α-induced cell–ECM adhesion, as shown by reduced FAK staining (Fig. 7e and f). Colocalization of activated β1 integrin and FAK was noted only in the cell periphery after pUR4 incubation (Fig. 7e, f, h, i, and k). Endothelial cells of CNS vasculatures also expressed αvβ3, another integrin receptor for FN [14]. TNF-α stimulation significantly depleted β3 integrin expression compared with that in unstimulated cells (Additional file 1: Figure S5a, b, and d), and the addition of pUR4 did not restore the expression of β3 integrin to the basal level (Additional file 1: Figure S5c and d), suggesting that the effects of pUR4 on the reduction of TNF-α-induced endothelial hyperpermeability may not be related to β3 integrin–mediated signaling.

**Fig. 8:**
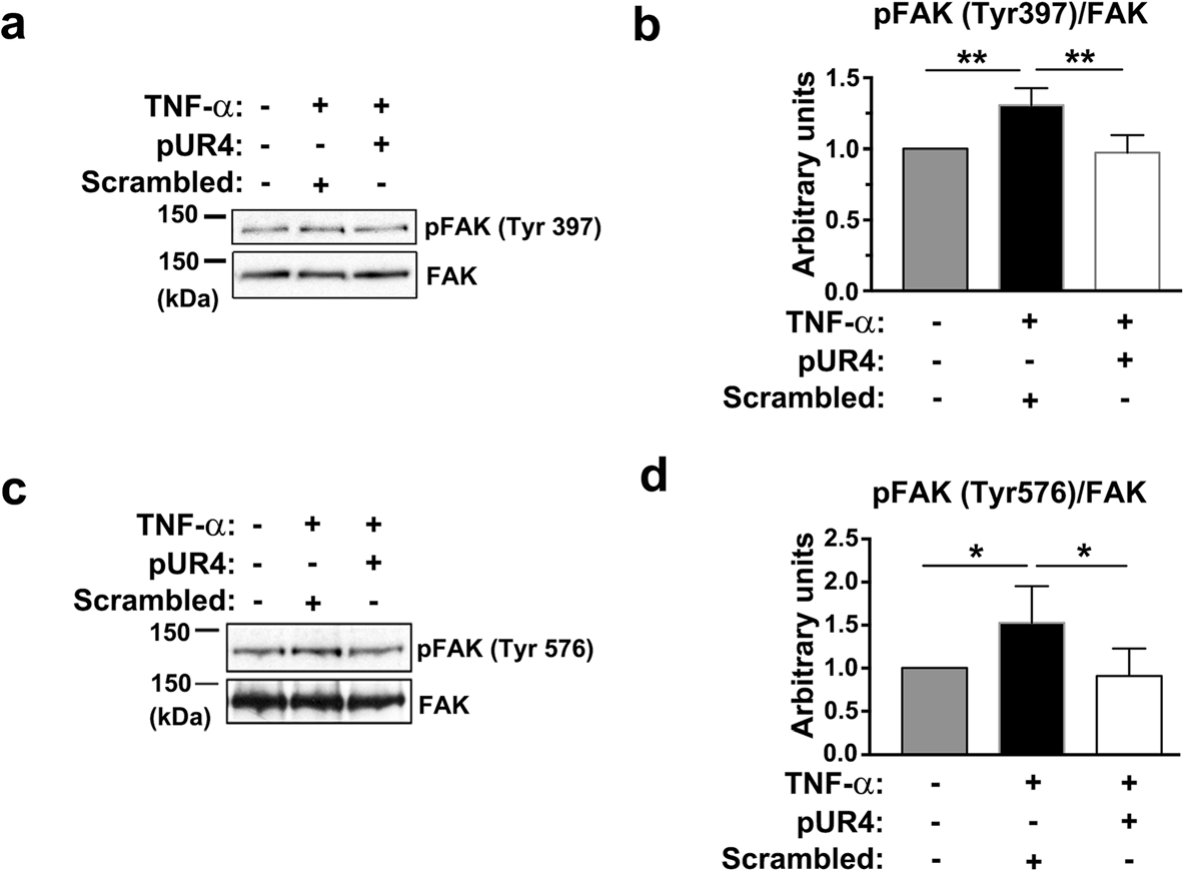
TNF-α-induced FAK phosphorylation is decreased by pUR4. After being pretreated with 1000 nM scrambled peptide or 1000 nM pUR4 for 16 h, bEND.3 cells were stimulated with TNF-α (20 ng/mL) for 6 h. FAK phosphorylation at Tyr-397 (**a**) and Tyr-576 (**c**) were determined through immunoblotting. Quantitative analysis of phosphorylated FAK at Tyr-397 (**b**) and Tyr-576 (**d**) normalized to total FAK (*n* = 4). Data are represented as means ± standard deviations. *P < 0.05 and **P < 0.01, one-way ANOVA followed by Tukey’s multiple comparison test

We next determined whether FN inhibition with pUR4 reduces β1 integrin–mediated downstream signaling. FAK is a nonreceptor protein tyrosine kinase; in response to integrin clustering, FAK autophosphorylates at Tyr397 and in turn recruits Src family kinases, which phosphorylate FAK at Tyr576 and Tyr577, leading to increased activity of FAK [8, 57]. We then examined whether inhibition of FN by pUR4 attenuates β1 integrin–mediated FAK activation in TNF-α-treated bEND.3 cells. FAK activation was evaluated by immunoblotting with site-specific antiphosphorylated FAK antibodies. FAK phosphorylation at Tyr397 and Tyr576 increased after 6 h of TNF-α treatment (Fig. 8). By contrast, the addition of pUR4 significantly attenuated FAK phosphorylation at Tyr397 and Tyr576 compared with that in the bEND.3 cells incubated with scrambled peptide (Fig. 8). Taken together, these data suggest that inhibition of FN deposition with pUR4 blocks TNF-α-mediated endothelial disruption by reducing FN–β1 integrin coupling and downstream signaling cascades.

## Discussion

Consistent with published in vitro and in vivo data, our study revealed that pUR4 efficiently blocked excessive FN deposition into the ECM of TNF-α-treated bEND.3 cells (Fig. 2). Moreover, in the TNF-α-induced inflamed endothelial monolayer, pUR4 was an effective inhibitor of TNF-α-induced compromised endothelial integrity (Fig. 3). pUR4 reduced TNF-α-induced cell morphology alteration (Fig. 5), actin stress fiber formation (Fig. 6), and actomyosin interaction (Fig. 6), leading to the attenuation of paracellular gap formation. Additionally, pUR4 reduced TNF-α-induced activation of β1 integrin (Fig. 7) and downstream signaling (Fig. 8).

We demonstrated that excessive FN deposited by endothelial cells under the pathological condition disrupted endothelial integrity. In addition to FN being secreted by endothelial cells, it can be derived from other sources. Plasma FN, which is secreted by hepatocytes and circulates in the blood [58–61], may extravasate from the compromised BSCB and be incorporated into the ECM around the microvessels and parenchyma of the spinal cord after peripheral nerve injury [37, 62]. Moreover, cellular FN may be locally produced by infiltrated macrophages in the CNS [63] as well as by astrocytes, another component of the BSCB activated in the injured spinal cord [64–66]. However, whether astrocyte-derived FN regulates the permeability of microvessels in the CNS has yet to be explored. An in vitro BBB or BSCB coculture system with endothelial cells and astrocytes could aid in understanding the interplay between accumulated astrocyte FN and endothelial permeability.

In this paper, we note that pUR4 blocked the deposition of ECM FN and then reduced pathological endothelial leakage by inhibiting paracellular gap formation. FN is secreted out of cells in soluble form and incorporated into the ECM as insoluble fibrils around the cells [67]. ECM FN is functionally distinct from soluble protomeric FN [68, 69]. Moreover, ECM FN promotes neovessel formation by inducing β1 integrin–mediated endothelial tractional forces, thereby causing extensive disruption of the cell–cell junction complex during angiogenesis [44]. Corroborating this result, the present study demonstrated that deposition of ECM FN around endothelial cells promoted the disruption of cell–cell adhesion through integrin ligation. By contrast, some studies have indicated that the application of soluble FN significantly reduces endothelial leakage both in vitro and in vivo [70]. This discrepancy between our study and aforementioned studies further supports the concept that the ECM form of proteins possesses properties distinct from soluble proteins.

The endothelial lining of CNS vessels predominantly expresses β1 integrin, which is associated with CNS vasculature development and maintenance. During CNS development, β1 integrin expression is considerably upregulated in endothelial cells, which regulate blood vessel formation [11]. Although β1 integrin expression is downregulated in endothelial cells after vessel maturation, β1 integrin maintains cell–cell junction integrity by stabilizing VE-cadherin and claudin-5 [3, 71]. The present study showed the importance of intact FN in the maintenance of normal endothelial integrity by demonstrating that pUR4 led to endothelial leakage in the unstimulated situation. However, the interaction between cells and the ECM was altered under the pathological condition, and CNS insults can strongly reinduce β1 integrin expression in endothelial cells [12]; furthermore, β1 integrin–mediated signaling can promote vascular leakage during inflammation [13, 56]. Our and other laboratories have reported that CNS insults significantly upregulate FN expression in addition to that of β1 integrin in the endothelial cells of microvessels [12]; thus, increased FN–β1 integrin interaction may drive endothelial barrier disruption. These data increase the possibility that decoupling of FN and β1 integrin may have therapeutic potential for leaky endothelial barriers. In this study, the addition of the FN inhibitor pUR4 attenuated β1 integrin activation and maintained the monolayer barrier function of bEND.3 cells. A 49-mer synthetic peptide, namely pUR4, can efficaciously penetrate animal tissues, as verified previously [26]. Our current results suggested therapeutic potential of pUR4 for endothelial leakage due to inflammatory diseases or injury in patients.

## Conclusions

This study employed the FN inhibitor pUR4 to identify the importance of ECM FN as a mediator in TNF-α-induced endothelial monolayer hyperpermeability. The results revealed that pUR4 prevented excessive FN deposition in the inflamed endothelial monolayer. Moreover, pUR4 disrupted FN–β1 integrin coupling and attenuated TNF-α-induced stress fiber formation, actomyosin interaction, and paracellular gap formation, suggesting that pUR4 can serve as a novel intervention for treating pathological vascular leakage.

## Additional file

Additional file 1: **Figure S1.** High pUR4 dose disrupts ECM FN fibrils assembled by bEND.3 cells. **Figure S2.** High pUR4 dose impairs bEND.3 cell adhesion and monolayer integrity. **Figure S3.** TNF-α-induced monolayer hyperpermeability in human cerebral microvascular endothelial (hCMEC/D3) cells is prevented by pUR4. **Figure S4.** VE-cadherin expression is not altered by pUR4. **Figure S5.** β3 integrin expression is not restored by pUR4. (DOCX 1681 kb)

## Abbreviations

BBB: Blood–brain barrier
bEND.3 cells: brain-derived endothelial cells
BSCB: Blood–spinal cord barrier
CNS: Central nervous system
ECM: Extracellular matrix
FAK: Focal adhesion kinase
FI: Fluorescence intensity
FITC: Fluorescein isothiocyanate
FN: Fibronectin
GAPDH: Glyceraldehyde 3-phosphate dehydrogenase
hCMEC/D3 cells: Human cerebral microvascular endothelial cells
MLC: Myosin light chain
PBS: Phosphate-buffered saline
PCR: Polymerase chain reaction
TEER: Transendothelial electrical resistance
TNF-α: Tumor necrosis factor-α
VE-cadherin: Vascular endothelial-cadherin
