# Supplementary material for: Fibronectin inhibitor pUR4 attenuates tumor necrosis factor α–induced endothelial hyperpermeability by modulating β1 integrin activation
Source: J Biomed Sci. 2019 May 16;26:37. doi: 10.1186/s12929-019-0529-6 (PMC6521375; doi:10.1186/s12929-019-0529-6)
Supplement: Supplementary file 1 — Figure S1. High pUR4 dose disrupts ECM FN fibrils assembled by bEND.3 cells. Figure S2. High pUR4 dose impairs bEND.3 cell adhesion and monolayer integrity. Figure S3. TNF-α-induced monolayer hyperpermeability in human cerebral microvascular endothelial (hCMEC/D3) cells is prevented by pUR4. Figure S4. VE-cadherin expression is not altered by pUR4. Figure S5. β3 integrin expression is not restored by pUR4. (DOCX 1681 kb) [file 12929_2019_529_MOESM1_ESM.docx]

**
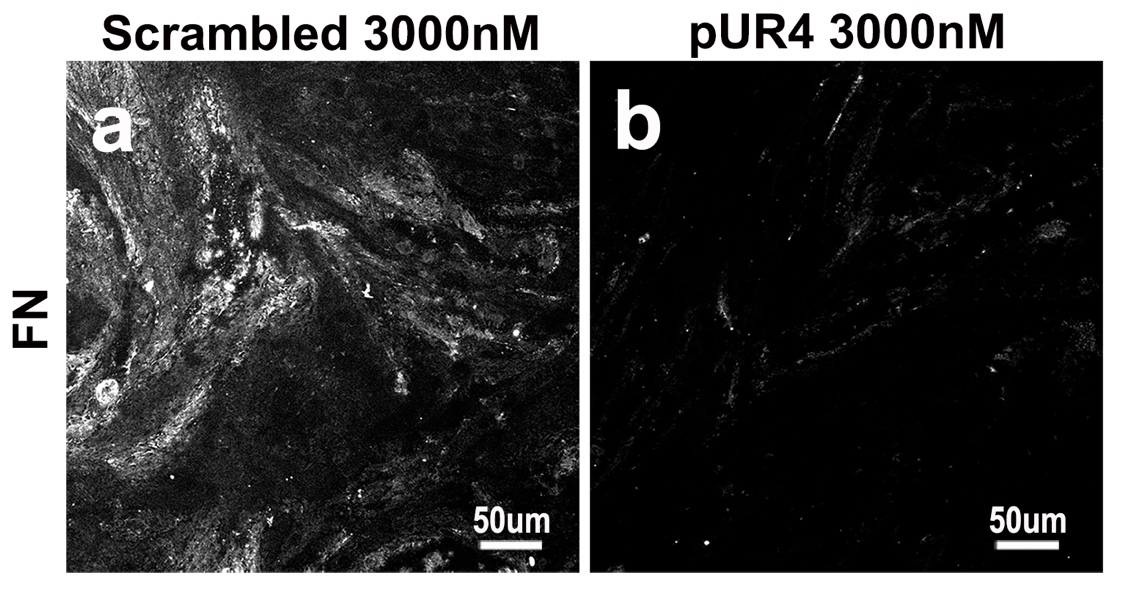
**

**Figure S1.** High pUR4 dose disrupts ECM FN fibrils assembled by bEND.3 cells. Samples of bEND.3 cells were incubated with 3000 nM scrambled peptide (a) or 3000 nM pUR4 (b) for 16 h. (a)–(b) Immunofluorescence analysis for FN was performed on nonpermeabilized bEND.3 cells to evaluate FN fibrils accumulated outside the cells. Confocal laser scanning photomicrographs revealed a considerable decrease in ECM FN assembled by bEND.3 cells treated with a high pUR4 dose.

**
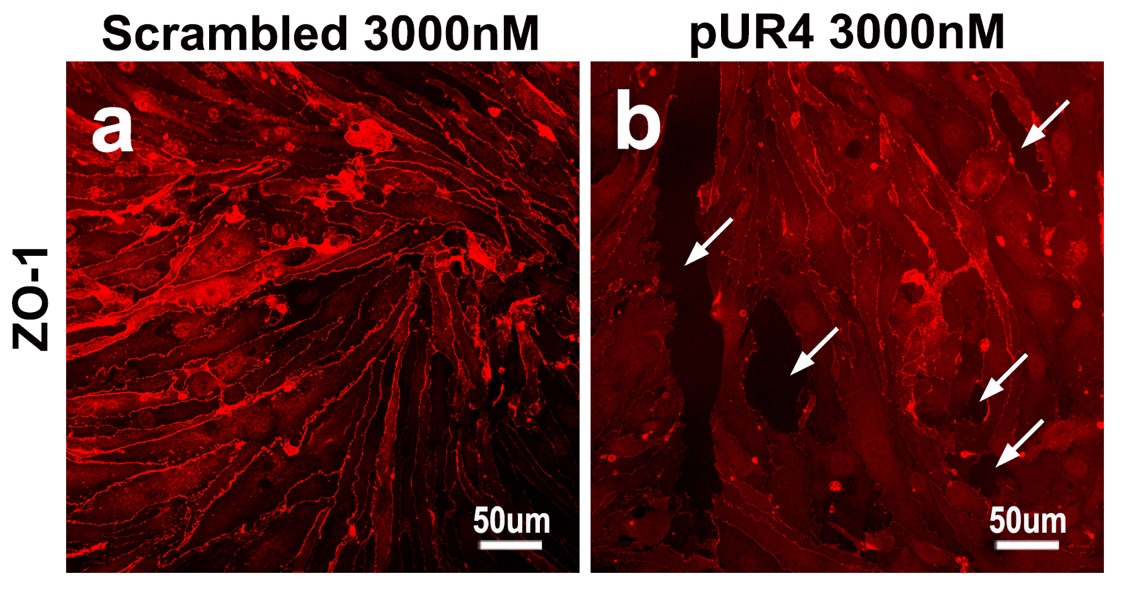
**

**Figure S2.** High pUR4 dose impairs bEND.3 cell adhesion and monolayer integrity. Samples of bEND.3 cells were incubated with 3000 nM scrambled peptide (a) or 3000 nM pUR4 (b) for 16 h. The cells were immunostained with anti-ZO-1 antibody to depict the cell boundaries. (a) The high scrambled peptide dose did not alter the endothelial integrity. (b) The high pUR4 dose disrupted the continuous pattern of ZO-1; some cells were even detached (indicated by arrows).

**
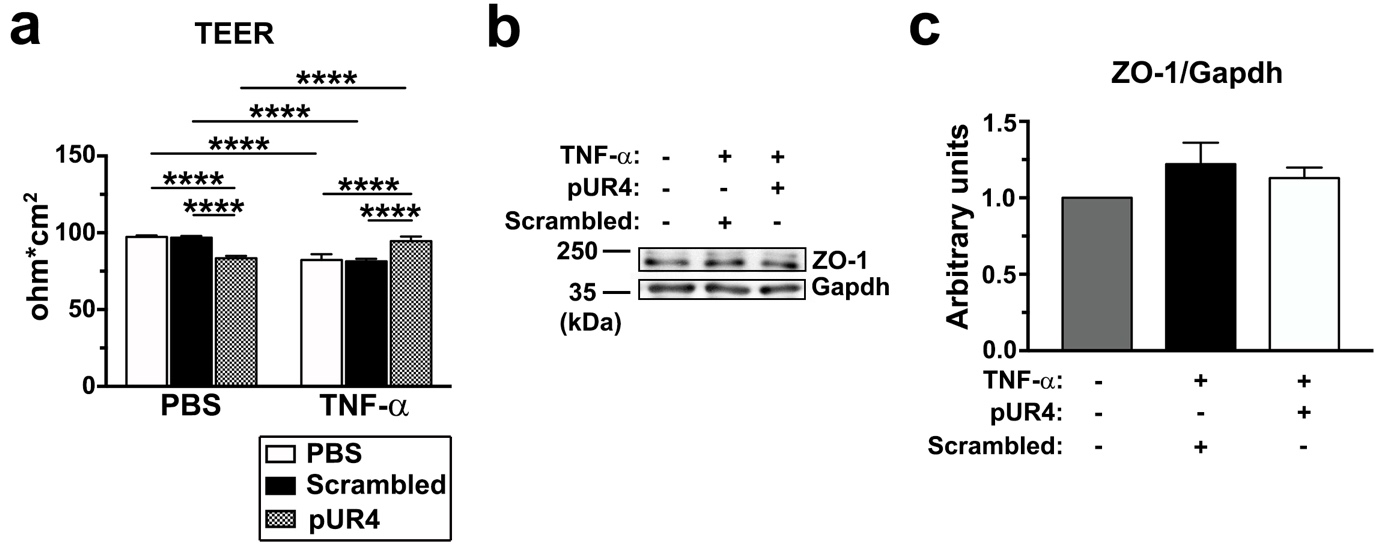
**

**Figure S3**. TNF-α-induced monolayer hyperpermeability in human cerebral microvascular endothelial (hCMEC/D3) cells is prevented by pUR4. After being grown on Transwell inserts, hCMEC/D3 cells were pretreated with 1000 nM scrambled peptide, 1000 nM pUR4, or PBS for 16 h and then underwent incubation with 20 ng/mL TNF-α or PBS control for 24 h. (a) TEER assay was performed 24 h after TNF-α or PBS treatment to determine hCMEC/D3 monolayer integrity (n = 6 for each experimental group). Data are presented as means ± standard deviations. *****P* < 0.0001, two-way ANOVA followed by Tukey’s multiple comparison test and Sidak’s multiple comparison test. (b) and (c) After being pretreated with 1000 nM scrambled peptide or 1000 nM pUR4 for 16 h, hCMEC/D3 cells were stimulated by 20 ng/mL TNF-α for 24 h. The protein expression of ZO-1 was evaluated through immunoblotting (b). Quantitative analysis of ZO-1 normalized to GAPDH is shown (c). No evident differences were observed among the experimental groups.

**
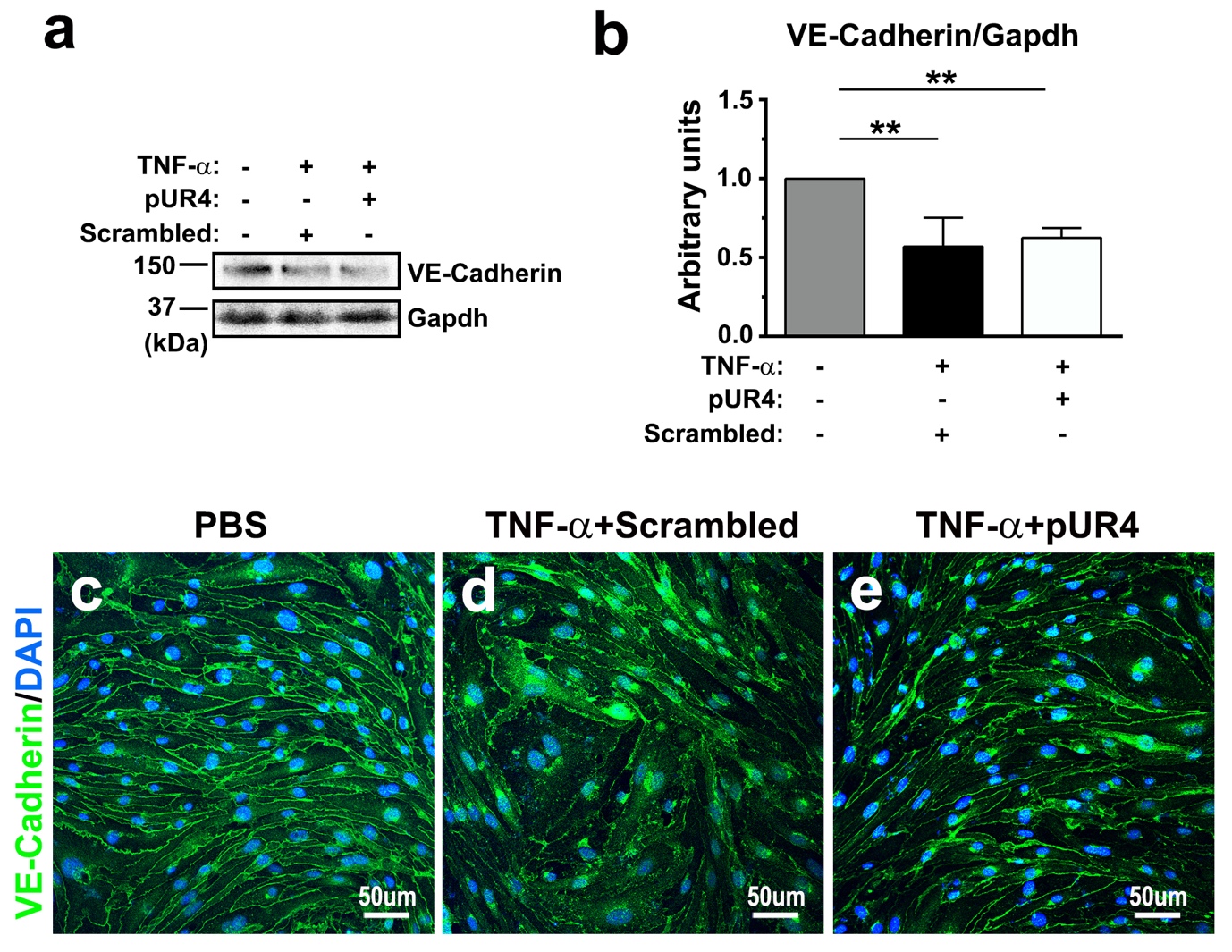
**

**Figure S4.** VE-cadherin expression is not altered by pUR4. After being preincubated with 1000 nM scrambled peptide or1000 nM pUR4 for 16 h, bEND.3 cells were exposed to TNF-α for 24 h. (a) The protein expression of VE-cadherin was evaluated through immunoblotting. (b) Quantitative analysis of VE-cadherin normalized to GAPDH is shown. (c)–(e) Expression of VE-cadherin was evaluated through immunofluorescence analysis. TNF-α induced a discontinuous pattern of VE-cadherin in the cell border (d). The addition of pUR4 did not significantly restore VE-cadherin integrity in the cell–cell junctions (e).


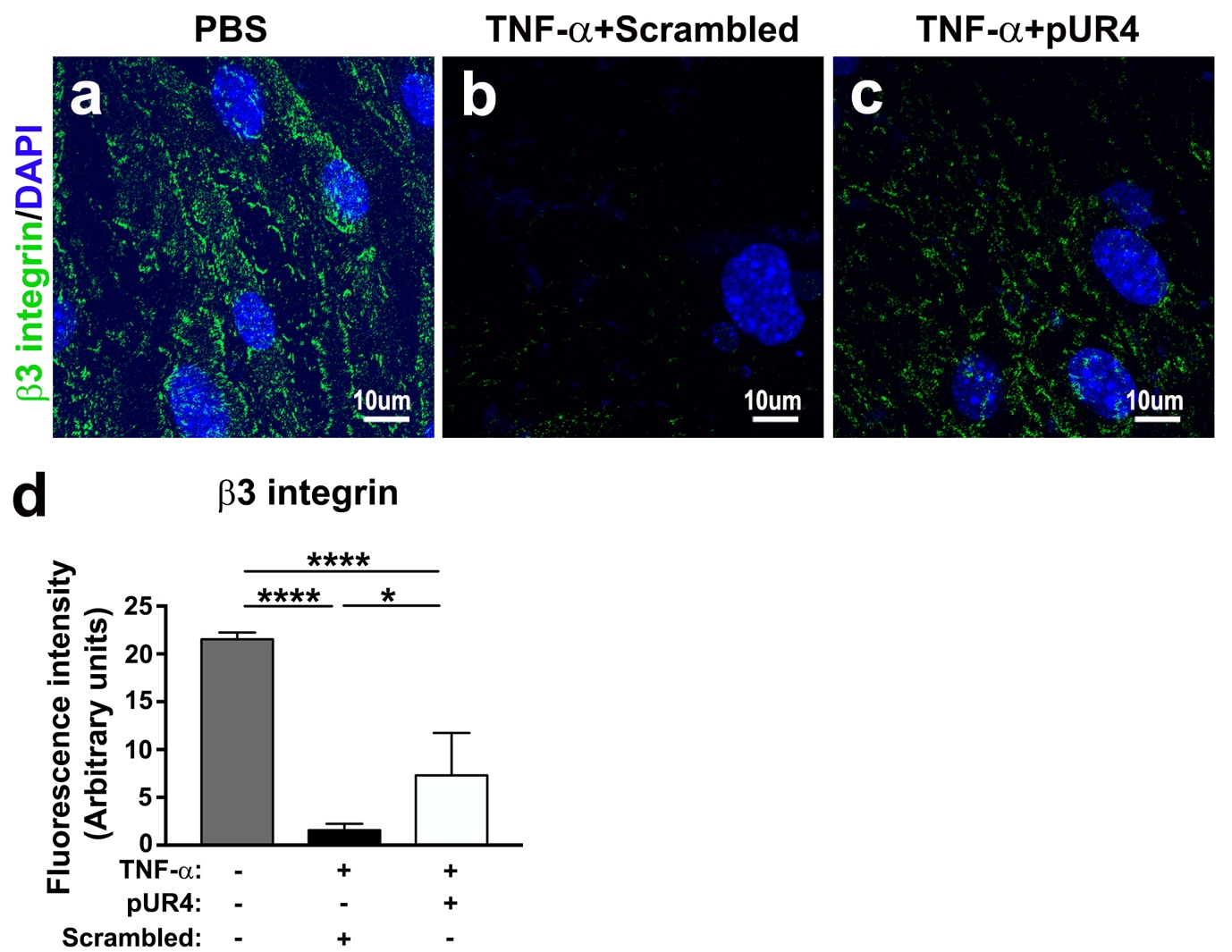


**Figure S5.** β3 integrin expression is not restored by pUR4. After being preincubated with 1000 nM scrambled peptide or 1000 nM pUR4 for 16 h, bEND.3 cells were treated with TNF-α for 24 h. PBS-treated control and TNF-α-treated bEND.3 cells were immunostained with anti-β3 integrin antibody. Three independent experiments were performed, and each experiment was repeated with similar results. (a)–(c) Representative immunofluorescence images showing β3 integrin in control and bEND.3 cells treated with the scrambled peptide and pUR4. The fluorescence intensity for β3 integrin was measured in a representative experiment (n = 3; d). Data are represented as means ± standard deviations. **P* < 0.05 and *****P* < 0.0001, one-way ANOVA followed by Tukey’s multiple comparison test.
